# Systemically identifying and prioritizing risk lncRNAs through integration of pan-cancer phenotype associations

**DOI:** 10.18632/oncotarget.14510

**Published:** 2017-01-05

**Authors:** Chaohan Xu, Rui Qi, Yanyan Ping, Jie Li, Hongying Zhao, Li Wang, Michael Yifei Du, Yun Xiao, Xia Li

**Affiliations:** ^1^ College of Bioinformatics Science and Technology, Harbin Medical University, Harbin, Heilongjiang, China; ^2^ Key Laboratory of Cardiovascular Medicine Research, Harbin Medical University, Ministry of Education, China; ^3^ Weston High School of Massachusetts, Massachusetts, USA

**Keywords:** disease phenotype association, risk lncRNA, pan cancer, identification and prioritization

## Abstract

LncRNAs have emerged as a major class of regulatory molecules involved in normal cellular physiology and disease, our knowledge of lncRNAs is very limited and it has become a major research challenge in discovering novel disease-related lncRNAs in cancers. Based on the assumption that diverse diseases with similar phenotype associations show similar molecular mechanisms, we presented a pan-cancer network-based prioritization approach to systematically identify disease-specific risk lncRNAs by integrating disease phenotype associations. We applied this strategy to approximately 2800 tumor samples from 14 cancer types for prioritizing disease risk lncRNAs. Our approach yielded an average area under the ROC curve (AUC) of 80.66%, with the highest AUC (98.14%) for medulloblastoma. When evaluated using leave-one-out cross-validation (LOOCV) for prioritization of disease candidate genes, the average AUC score of 97.16% was achieved. Moreover, we demonstrated the robustness as well as the integrative importance of this approach, including disease phenotype associations, known disease genes and the numbers of cancer types. Taking glioblastoma multiforme as a case study, we identified a candidate lncRNA gene SNHG1 as a novel disease risk factor for disease diagnosis and prognosis. In summary, we provided a novel lncRNA prioritization approach by integrating pan-cancer phenotype associations that could help researchers better understand the important roles of lncRNAs in human cancers.

## INTRODUCTION

Long noncoding RNAs (lncRNAs) are a class of non-protein coding transcripts that are longer than 200 nucleotides [1]. They regulates key cellular processes due to their roles in DNA and RNA metabolism and are involved in many complex human diseases including cancer [2, 3]. Systematic studies using high-throughput molecular tools have identified more than 12000 lncRNAs encoded in the human genome with little or no protein-coding capacity (GENCODE Release 23). Cumulative evidence suggests that lncRNAs play crucial roles in tumorigenesis and metastasis. Some lncRNAs, similar to protein-coding genes, can be considered as “oncogenes” or “tumor suppressors” for cancers and are valuable in cancer diagnosis and prognosis [4–7]. However, despite enormous progress made by high-throughput biological detection techniques, the identification of disease related lncRNAs has remained a great challenge for researchers.

Several computational approaches have been proposed to infer novel relationships between lncRNAs and diseases [8–15]. Zhou *et al*. proposed a novel rank-based method (RWRHLD) to prioritize candidate lncRNAs [12]. They constructed a miRNA-associated lncRNA crosstalk network by considering significant co-occurrence of miRNA response elements (MREs) on lncRNA transcripts, a disease–disease similarity network by a directed acyclic graph (DAG) structure and a lncRNA-disease network by using experimentally confirmed lncRNA–disease associations obtained from lncRNADisease. They integrated these three networks into a heterogeneous network and implemented a random walk with restart on the network for prioritizing candidate disease lncRNAs. They used leave-one-out cross-validation to test the performance of this method based on known experimentally verified lncRNA–disease associations and predict several novel lncRNA–disease associations predicted in ovarian cancer and prostate cancer. In our recent work, we proposed a computational method based on naive Bayesian to identify cancer-related lncRNAs by integrating genome, regulome and transcriptome according to known disease lncRNAs [13]. We totally identified 707 potential cancer-related lncRNAs and demonstrated the performance of the method by ten-fold cross-validation. We found that integration of multi-omic data was necessary to identify cancer-related lncRNAs and our results showed that these candidate lncRNAs tend to exhibit significant differential expression and differential DNA methylation in multiple cancer types. However, the limitation of these studies was the relatively small number of known disease lncRNAs. Subsequently, to improve the limitation, some studies have used known disease miRNAs to help infer disease lncRNAs [12, 14]. Chen *et al*. developed a computational model named Hyper Geometric distribution for LncRNA-Disease Association inference (HGLDA) to prioritize disease candidate lncRNAs [14]. Based on known miRNA-disease relations and experimentally confirmed lncRNA-miRNA interactions detected by CLIP-Seq technology, they used hypergeometric distribution test for each lncRNA-disease pair to detect whether they significantly shared common miRNAs. Those lncRNA-disease pairs with FDR less than 0.05 were selected to be potential lncRNA-disease associations. Moreover, they developed the LFSCM (LncRNA Functional Similarity Calculation based on the information of MiRNA) model to calculate large-scale lncRNA functional similarity by integrating disease semantic similarity, miRNA-disease associations, and miRNA-lncRNA interactions.

Disease associations (namely disease phenotype similarities) can be used to improve the limitation by complementary disease information, which has been successfully applied in prioritization of disease protein-coding genes and miRNAs [16–18]. These studies hypothesized that highly phenotype similar diseases tend to show more close relations, and their relevant genes or miRNAs often reside in the same neighborhood in the interaction networks and form physical or functional modules. Although individual disease may contain only a few information, combination of multiple phenotype similar diseases based on the assumption can provide many additional clues as for the specific disease. Even for those diseases without any known information, such disease association hypothesis can help to reveal some potential risk factors. In our own previous work [17], we presented a miRNA prioritization approach to identify disease-specific miRNAs by using known disease genes and context-dependent miRNA-target interactions derived from matched miRNA and mRNA expression data, independent of known disease miRNAs. Further, we applied this approach to systematically prioritize miRNAs involved in 11 cancer types and yielded an average AUC value of 75.84% based on known disease miRNAs. Due to insufficient disease information on lncRNAs, it is imperative to identify disease-related lncRNAs by curating disease associations or disease knowledge of other risk factors. Additionally, a large number of array-based expression datasets were produced during the past two decades. These array-based expression datasets that have less technical variation and better detection sensitivity can be re-annotated to interrogate lncRNA expression changes when dealing with low-abundance transcripts [19–24]. Array-based datasets simultaneously capture and monitor gene and lncRNA expression in the same cancer samples for diverse cancer types, improves the confirmation process and the quality of identifying lncRNA related genes in the specific contexts [25–27].

Therefore, the aim of our study was to generate an lncRNA computational approach to systematically prioritize and identify candidate disease risk lncRNAs by integrating disease phenotype associations. We interrogated lncRNA expression in thousands of tumor samples and constructed a gene and lncRNA co-expression pan-cancer network (GLCPN) for 14 cancer types. Utilization of known disease genes as seeds independently of disease lncRNAs, we used random walk method to prioritize candidate disease lncRNAs for each cancer type. The average AUC score is 80.66% for prioritization of candidate disease lncRNAs and 97.16% for protein-coding genes. Our results show that through the integration of disease phenotype associations, the lncRNA prioritization performance can be improved, especially for some diseases with few or without known disease lncRNAs.

## RESULTS

### Construction of GLCPN using the pan-cancer data

Through comprehensively searching “Affymetrix Human Exon 1.0 ST array” in GEO and ArrayExpress databases, forty-three array-based expression studies consisting of 2828 disease samples from fourteen cancer types were identified for our study. The cancer types included bladder cancer (BLC), breast cancer (BC), hepatocellular carcinoma (HCC), gastric cancer (GC), glioblastoma multiforme (GBM), renal cell carcinoma (RCC), medulloblastoma (MB), melanoma (MM), prostate cancer (PC), lung cancer (LC), lymphoblastic leukemia (LL), neuroblastoma (NB), cervical cancer (CC) and ovarian cancer (OC) (Supplementary Table 1). Through re-annotation, 18376 unique genes and 10092 lncRNAs covered by at least four probes were obtained (Supplementary Figure 1A). After repurposing the expression datasets to probe lncRNA expression for each cancer study, lncRNA expression datasets that had the same number of disease samples as the gene expression datasets were generated. We found that the expression levels of lncRNAs in fourteen cancer types were generally lower than genes (Supplementary Figure 1B), which is consistent with previously reported re-annotation studies [21, 25, 28].

**Figure 1:**
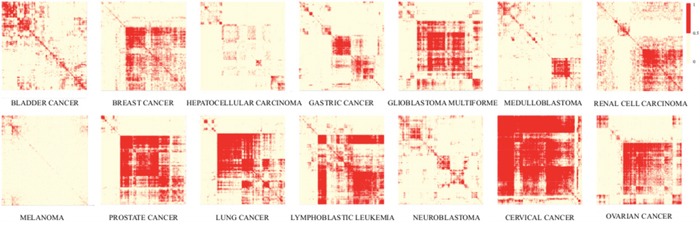
Heat maps of co-expression relationships in the fourteen cancer-types Clustering maps showing existence of gene-gene or lncRNA-gene co-expression relationships among different cancer types that can be used to construct disease-specific sub-networks.

Based on the assumption that genes and lncRNAs with similar expression have similar functions, we constructed disease-specific co-expression sub-networks for each cancer type according to the gene-gene and lncRNA-gene co-expression associations (Figure 1). These fourteen sub-networks were further integrated into a GLCPN. Protein interactions derived from STRING database were also incorporated into the GLCPN. Finally, the pan-cancer network that was constructed included 29071 nodes and 159132861 edges (Supplementary Table 2). The co-expression frequency in the fourteen cancer types or the protein interaction probability obtained from the STRING database was used to weight the edges. This weighted functional network was used for the following prioritization of risk lncRNAs.

### Prioritization of disease risk lncRNAs by integrating of disease phenotype associations

To efficiently prioritize candidate disease lncRNAs, we proposed a method based on the random walk that used known disease genes and phenotype similarities to quantify the links between known disease genes and candidate disease lncRNAs in the GLPCN. For one cancer type, a prediction score for each candidate lncRNA was computed (see Methods). Finally, fourteen candidate lncRNA lists represented the prioritization results of fourteen cancer types were generated.

**Figure 2:**
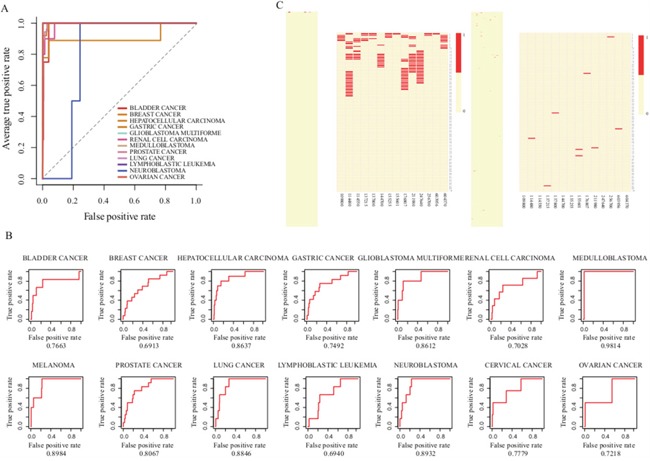
Evaluation of the performance of our lncRNA prioritization approach **A**. The ROC curves of gene prioritization results by LOOCV **B**. The ROC curves of lncRNA prioritization results. **C**. Top 100 ranks of known disease genes (left) and lncRNAs (right) after prioritization.

To further investigate the performance of our approach in prioritization of genes, we then performed the LOOCV analysis. Since only one known disease gene respectively can be found in CC and MM, we applied LOOCV to other twelve expression studies. The average AUC score of twelve cancers can reach 97.16%, strongly supporting that our prioritization approach has good prioritization performance (Figure 2A). During the leave-one-out cross validation, we found that all known disease genes in twelve cancer types were ranked in the top 40 out of 18979 genes (0.22%) in the corresponding candidate disease gene lists. For example, known disease genes *BRCA2, CDH1, IDH1, CDKN2A, NME1* and *FGFR3* frequently occurred at the top one in seven cancer types (including BC, GC, GBM, MB, MM, NB and OC), even though only two or three known-disease genes existed in NB, GBM and MB gene lists. To further evaluate the performance of our approach in prioritization of lncRNAs, we extracted known disease lncRNAs of the fourteen cancer types from the LncRNADisease database and computed their AUC scores. The average AUC of fourteen cancers was 80.66% (Figure 2B), suggesting that our methodology efficiently prioritized and identified cancer related risk lncRNAs.

Furthermore, we also investigated the overall distribution of known disease lncRNAs at the top of candidate lists (Figure 2C). Some recently identified disease lncRNAs like *MYCNOS, CDKN2B-AS1, WT1-AS, IGF2-AS* and *GAS5* that play important roles in NB, GBM, LL, RCC and PC [29–33], were ranked at the top of the candidate lists. Intriguingly, we found that more than half of the known disease lncRNAs were ranked at the top 10% of prioritization lists in ten cancer types, namely, BC, HCC, GBM, MB, MM, PC, LC, NB, CC and OC. Moreover, we also selected five representative disease lncRNAs (*HOTAIR, MALAT1, H19, MEG3* and *TUG1*) that play key roles as oncogenic molecules associated with various cancers [34–37] to investigate their occurrences at the top 5%-20% of the candidate lists (Supplementary Figure 2A). We found that *HOTAIR, H19* and *TUG1* almost appeared in all cancer types.

### Evaluation of the robustness and the integration importance of lncRNA prioritization approach

The principle of our lncRNA prioritization approach depended on the disease phenotype associations among diverse cancer types, as well as the topological similarities between known disease genes and context-specific co-expression genes of lncRNAs in the GLCPN. Therefore, it was important to evaluate the contribution of these factors to the performance of our lncRNA prioritization approach.

### Evaluation of the influence of disease phenotype associations

**Figure 3:**
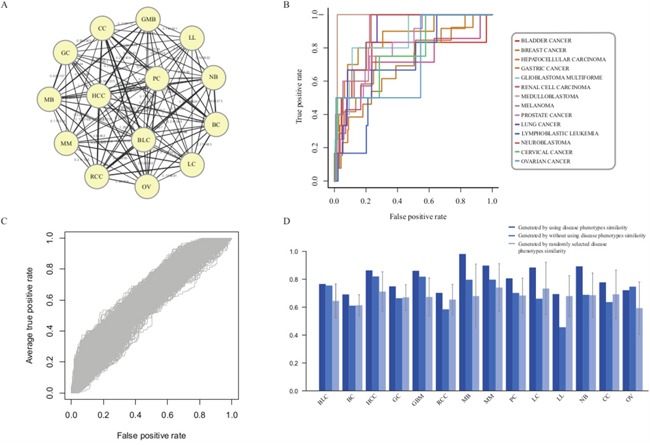
Evaluation of the influence of disease phenotype associations **A**. Fourteen disease phenotype association network. **B**. The ROC curves of lncRNA prioritization results generated by excluding disease phenotype associations. **C**. The ROC curves generated by randomly selecting disease phenotype associations with 1000 repetitions. **D**. The comparison results of lncRNA prioritization generated by either using, excluding or permuting disease phenotype associations.

The fourteen disease phenotype associations can be used to characterize the relationship between diseases and provide the opportunity for us to elucidate the pathogenesis mechanisms of diseases in the crosstalk pattern (Figure 3A and Supplementary Table 3). To evaluate the importance of disease phenotype associations, we prioritized risk lncRNAs in diverse cancer types without utilizing any phenotype associations. The average AUC based on known disease lncRNAs from LncRNADisease was 78.78%, lower than the AUC score (80.66%) with the inclusion of disease phenotype associations (Figure 3B and 3D). Notably, the AUC score for LL dropped from 69.4% to 45.73%, suggesting that the disease phenotype associations can be efficiently used to supply the incomplete information of some diseases and improve the overall performance of lncRNA prioritization.

To further evaluate the influence of disease phenotype similarity, we permuted the phenotype similarity matrix and recomputed the prioritized scores for all candidate lncRNAs (Figure 3C and 3D). The average AUC score was 61.21%. Taken together, the decreased performances in prioritization by remove or permute phenotype associations supported that the disease phenotype association was one of necessary and indispensable factors for the lncRNA prioritization in our approach. It also showed that the disease phenotype associations could efficiently complement the incomplete disease information in individual cancer types and thus provide more power to identify cancer-related lncRNAs through pan-cancer analysis.

### Evaluation of the influence of the number of cancer types

Disease phenotype associations can be used to bridge relationships among different cancer types and efficiently improve the performance of our prioritization approach. Therefore, we sought to determine the performance of our lncRNA prioritization approach by varying the number of cancer types. Towards this, we randomly selected 3, 6, 9 and 12 cancers from the original fourteen cancer types to re-compute prioritization scores for candidate disease lncRNAs. We found that upon increasing the number of cancer types for analysis, the average AUC scores increased from 73.49% to 80.01% (Supplementary Figure 2B). This suggested that utilization of more diseases with their phenotype associations can facilitate the improvement of prioritization of candidate disease lncRNAs.

### Evaluation of the influence of known disease genes

Our lncRNA prioritization approach only relied upon known disease related protein-coding genes without the requirement of known disease lncRNAs. Therefore, we evaluated the efficiency of known disease genes by randomly selecting the same number of non-disease-associated genes as known disease genes for each cancer type. Owing to the lack of the non-disease gene set, we obtained a total of 43899 human genes from the NCBI Gene database and 15229 known disease genes from the OMIM database. A non-disease gene set containing 28670 genes was then generated. Equal numbers of non-disease genes for each cancer were randomly selected 1000 times and used for prioritization. We obtained an average AUC score of 59.21% that was significantly lower than the prioritization result based on known disease genes (*p*<0.001).

### Evaluation of prediction performance of our prioritization approach

To assess the prediction performance of our lncRNA prioritization approach, we prioritized candidate disease lncRNAs for each cancer type only using information of the other cancer types through disease phenotype similarities. Surprisingly, the average AUC was 81.63%, supporting that our lncRNA prioritization approach has superior performance in predicting of potential risk lncRNAs (Supplementary Figure 2C). All of the validation results showed that our prioritization approach has a good ability in identification of known disease lncRNAs and genes (Supplementary Figure 2D), even for some diseases with little or without known disease information.

### A case study of GBM

**Figure 4:**
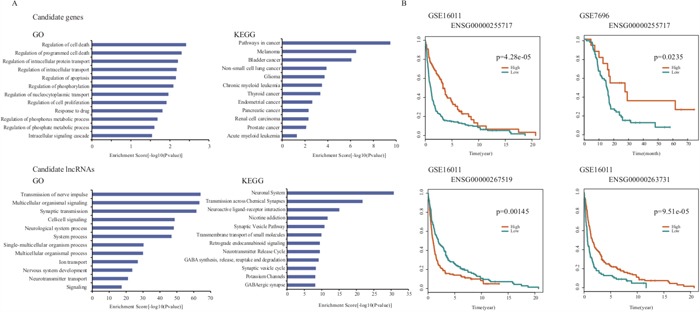
The prioritization results in the case study of GBM **A**. The GO and KEGG enrichment analysis results for top 17 non-disease candidate genes and top 20 candidate lncRNAs of GBM. **B**. Survival analysis results of three candidate lncRNAs in GSE7696 and GSE16011.

GBM is a highly aggressive brain cancer with extremely poor prognostic outcome despite intensive treatment regimes. Taking GBM as a case study, we used our approach to prioritize risk genes and lncRNAs associated with GBM. Through LOOCV, we found all known disease genes rank at the top of the candidate disease gene list, in which *IDH1, ERBB2*, and *TP53* are ranked at 1*^th^*, 2*^nd^* and 20*^th^*, respectively (Supplementary Table 4). We then extracted the top 20 candidate disease genes and carried out function enrichment analysis using the remaining seventeen unknown disease genes by DAVID (Benjamini test, *p*<0.01) [38]. We found that these candidate genes were significantly enriched in cancer-related GO functions, including “regulation of cell proliferation”, “regulation of apoptosis”, “regulation of programmed cell death”, “regulation of cell death” and “regulation of nucleocytoplasmic transport” (Figure 4A and Supplementary Table 5) and KEGG pathways, including “pathways in cancer”, “melanoma”, “bladder cancer”, “non-small cell lung cancer” and “glioma”. These results suggested that the seventeen candidate disease genes may play crucial roles in GBM tumorigenesis (Figure 4A and Supplementary Table 5).

For the lncRNA prioritization result, we extracted the top 5% of lncRNAs and found all known disease lncRNAs, *CDKN2B-AS1* and *H19*, in the candidate disease lncRNA list. To further validate GBM-related lncRNAs with high-confidence, we chose the top 20 lncRNAs and investigated their potential functions according to LncRNA2Function (Benjamini test, *p*=0.01, Supplementary Table 6). We found that the lncRNA-related genes were significantly enriched in GBM-related GO terms and KEGG pathways. The GO terms included “transmission of nerve impulse”, “multicellular organismal signaling”, “synaptic transmission”, “cell-cell signaling”, “neurological system process”, “system process” and “nervous system development” etc. (Figure 4A). The KEGG enrichment analysis included “neuronal system”, “transmission across chemical synapses”, “neuroactive ligand-receptor interaction”, “neurotransmitter release cycle” and “transmembrane transport of small molecules” pathways etc. (Figure 4A).

Next, we investigated whether these candidate lncRNAs were independent prognostic factors for survival. Towards this, we obtained two public expression datasets from the GEO database that contained 80 and 263 GBM samples (GSE7696 and GSE16011), respectively, and performed the same re-annotation process as described in Method. We found 2673 lncRNAs that were then subjected to survival analysis based on which we identified, three lncRNAs including *ENSG00000267519, ENSG00000255717* and *ENSG00000263731* that significantly correlated with survival of GBM (Figure 4B). Interestingly, high expression of the lncRNA gene *ENSG00000255717*, namely *SNHG1*, correlated with poor prognosis in both of the two datasets. Previously, Cao *et al*. identified abnormal expression of *SNHG1* in gastric cancer [39]. Our findings suggested that these potential lncRNAs may promote the development of GBM and could serve as novel prognostic markers for GBM, once verified.

## DISCUSSION

Although a large number of lncRNAs have been identified in the human genome over the past decade [20–22, 27]. Only a few lncRNAs have been verified to be associated with diseases. How to integrate different biological datasets to accurately predict risk lncRNAs has become a critical issue for understanding disease mechanisms at the lncRNA level.

Based on the assumption that different diseases with similar phenotype associations involve similar molecular mechanisms, several studies have demonstrated that disease phenotype associations can help link different diseases with common genes and/or miRNAs [17, 18, 21, 40]. Disease phenotype associations have been widely used to benefit the systematic identification of disease-related protein-coding genes or miRNAs and facilitate in-depth understanding of their pathogenesis in human cancers. In this study, we developed a prioritization approach that was based on disease phenotype associations and systematically identified disease risk lncRNAs through integration of large-scale array-based expression datasets. Through collecting and re-annotating Affymetrix Human Exon 1.0 ST array datasets, we obtained 2818 samples with matched gene and lncRNA expressions in fourteen cancer types. We then constructed a GLCPN by using the pan-cancer datasets and found that our prioritization strategy was efficient in identifying candidate disease lncRNAs apart from being cost-effective. Our prioritization results showed that the top ranked lncRNAs or genes have high probabilities of being bona fide disease-related lncRNAs or genes.

The majority of disease candidate lncRNA prioritization approaches utilized known disease lncRNA information to predict disease and lncRNA associations [8–11, 13, 15]. However, only a few disease-related lncRNAs have been identified, and this limited information results in incomplete training sets during prioritization and hence can influence the performance in previous lncRNA prioritization approaches. Some other lncRNA prioritization approaches were designed by integration of other information, such as predictive and experimentally validated lncRNA-miRNA interactions [12, 14]. Such information provided the additional ability to measure the relationships between lncRNAs and diseases. Notably, the numbers of these known biological associations are relatively limited. LncRNA–miRNA interactions experimentally confirmed by molecular biological technologies in starBase v2.0 database refer to 1114 lncRNAs and 132 miRNAs. Such incomplete information will limit the power in prioritizing or predicting lncRNAs that are potential associated with diseases. Relative to known disease lncRNAs and miRNAs, more disease protein-coding genes have been identified and confirmed. In contrast to previous methods [8–14], our method required only the knowledge of known disease genes for prioritization and did not depend on known disease-related lncRNAs. This enabled us to prioritize more comprehensive risk lncRNAs associated with a specific disease even in diseases without any known disease-related lncRNAs. Moreover, our approach was based on disease phenotype associations that can reduce the influence of the limited numbers of known disease genes and are effective in the prioritization of disease risk lncRNAs. The strategy utilized in our approach can help to advance the understanding of lncRNA function in cancer etiology. In summary, we presented an integrated prioritization approach for systematically prioritizing risk lncRNAs associated with human disease. This approach can be used to facilitate the identification of disease-related lncRNAs and to increase the understanding of lncRNA-mediated pathogenesis. Using our approach, we performed overall prioritization of the risk lncRNAs for fourteen cancer types, which provided testable hypotheses to guide further experiments.

## MATERIALS AND METHODS

### Array-based expression data collection and re-annotation

To satisfy the requirement that re-annotated lncRNAs have the broad coverage across the whole genome and microarray platforms designed by distinct expression studies are consistent, we selected “Affymetrix Human Exon 1.0 ST Array” as the research platform and collected this kind of expression datasets from GEO and ArrayExpress databases [41]. In order to ensure the sufficient sample size, we selected cancer studies with five or more disease samples to be considered and used for the further analysis. After widespread screening, forty-three studies with 2828 disease samples were identified. They were associated with fourteen cancer types namely, bladder cancer (BLC), breast cancer (BC), hepatocellular carcinoma (HCC), gastric cancer (GC), glioblastoma multiforme (GBM), renal cell carcinoma (RCC), medulloblastoma (MB), melanoma (MM), prostate cancer (PC), lung cancer (LC), lymphoblastic leukemia (LL), neuroblastoma (NB), cervical cancer (CC) and ovarian cancer (OC) (Supplementary Table 1).

### Construction of lncRNA expression through re-annotation

We applied a custom pipeline to re-annotate Affymetrix Human Exon 1.0 ST Array taking advantage of its huge amount of probes annotated to thousands of lncRNAs. The probe sequences were downloaded from the manufacturer's website (http://www.affymetrix.com) and were then uniquely mapped to the human genome (hg19) by Bowtie without mismatch. Through using BEDTools (http://code.google.com/p/bedtools), probes completely falling into exons of lncRNAs but without overlapping with protein-coding genes were remained. Expression values of one lncRNA gene detected by at least four probes were averaged. All the expression data was log2-transformed and uniformly normalized by the quantile normalization approach. Finally, lncRNA expression datasets were constructed for all cancer types.

### Construction of a GLCPN across pan-cancer datasets

Guilt by association implies that genes or lncRNAs with similar expression patterns under multiple experimental conditions have a high probability of sharing similar functions or being involved in common biological pathways [42, 43]. Therefore, we constructed a GLCPN from all the cancer datasets we had collected. We used the Pearson correlation coefficient to quantify the relations between or within the genes and the lncRNAs from the expression datasets in all the 14 cancer types. For each cancer type, gene-gene and gene-lncRNA pairs with co-expression scores greater than 0.8 and 0.7, respectively, were combined to formed a disease-specific network in each dataset as performed in previous studies [44–46]. Furthermore, we integrated all associated disease-specific networks into one GLCPN. The edge weights of GLCPN were assigned by the frequencies in the different cancer types. We further integrated protein-protein interaction (PPI) relationships (17649 nodes and 2079530 edges) obtained from a publicly available database STRING database [47] into the GLCPN. The normalized interaction probability scores obtained from the STRING database were considered as the weights.

### Prioritization of risk lncRNAs and genes through integration of disease phenotype associations

**Figure 5:**
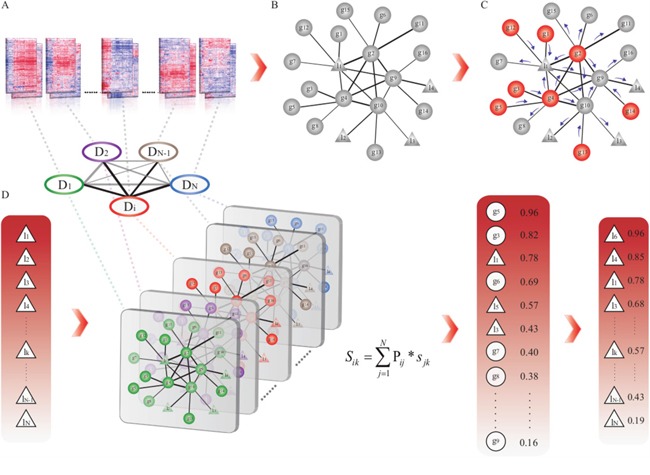
The workflow of prioritization of risk lncRNAs through integration of disease phenotype associations **A**. Array-based expression data collection and re-annotation. **B**. Construction of the gene and lncRNA co-expression pan-cancer network (GLCPN). **C**. Application of the random walk method to predict scores for all candidates according to known disease genes. **D**. Integration of prediction scores by disease phenotype associations and generation of disease candidate lncRNA lists for prioritization.

To efficiently prioritize risk lncRNAs and genes in different cancer types based on the GLCPN, we applied the random walk method to calculate prediction score for all candidate lncRNAs and genes in each cancer type. By considering the known disease genes as seed nodes for any queried cancer type ‘i’ (Figure 5), we utilized the random walk method to compute prediction scores for each node (gene or lncRNA) in the GLCPN. By assuming that diverse diseases with phenotype associations show similar molecular mechanisms, we further combined disease phenotype similarity scores with the prediction scores of lncRNAs or genes into a unique prioritization score S_ij_ by:

$$s_{ik}=\sum_{j=1}^{N}P_{ij}*s_{jk}$$

Where P_ij_ represents the disease phenotype similarity score between cancer type i and j, and S_jk_ represents the corresponding prediction score for candidate lncRNA (or gene) k in cancer type j (Figure 5). Disease phenotype similarity scores were derived from the MimMiner tool, which calculates the correlation scores of 5080 known disease phenotypes through text mining analysis [48]. The candidate disease genes and lncRNAs were then ranked according to the prediction scores.

### Evaluation of the robustness and the integration importance of our prioritization approach

We evaluated the performance of our prioritization approach by known disease lncRNAs and genes by using the ROC curve analysis, and the leave-one-out cross-validation (LOOCV) was carried out to assess the gene prioritization performance. Known causal genes and lncRNAs were extracted from the Online Mendelian Inheritance in Man (OMIM) [49] and the LncRNADisease database [50].

To evaluate the robustness and the integration importance of our prioritization approach, we accepted the evaluation strategies by leaving out or permuting relevant influence factors, included disease phenotype associations, the number of cancer types and known disease genes, and interrogated the changes in the prioritization results. Finally, we assessed the prediction performance of our prioritization approach in identifying disease-related lncRNAs and genes for each cancer type by only using information from other diseases.

### Gene or lncRNA functional enrichment analysis

Functional enrichment analysis of candidate genes was performed by using the DAVID bioinformatics tool (http://david.abcc.ncifcrf.gov/conversion.jsp). For lncRNAs, we used LncRNA2Function (http://mlg.hit.edu.cn/lncrna2function) to perform function characterization [51]. All statistical analyses were performed using the R software package (http://www.r-project.org).

## SUPPLEMENTARY MATERIALS FIGURES AND TABLES






