# Supplementary material for: Systemically identifying and prioritizing risk lncRNAs through integration of pan-cancer phenotype associations
Source: Oncotarget. 2017 Jan 5;8(7):12041–51. doi: 10.18632/oncotarget.14510 (PMC5355324; doi:10.18632/oncotarget.14510)
Supplement: Supplementary file 1 [file oncotarget-08-12041-s001.pdf]

Systemically identifying and prioritizing risk lncRNAs through integration of pan-cancer phenotype associations

SUPPLEMENTARY FIGURES AND TABLES

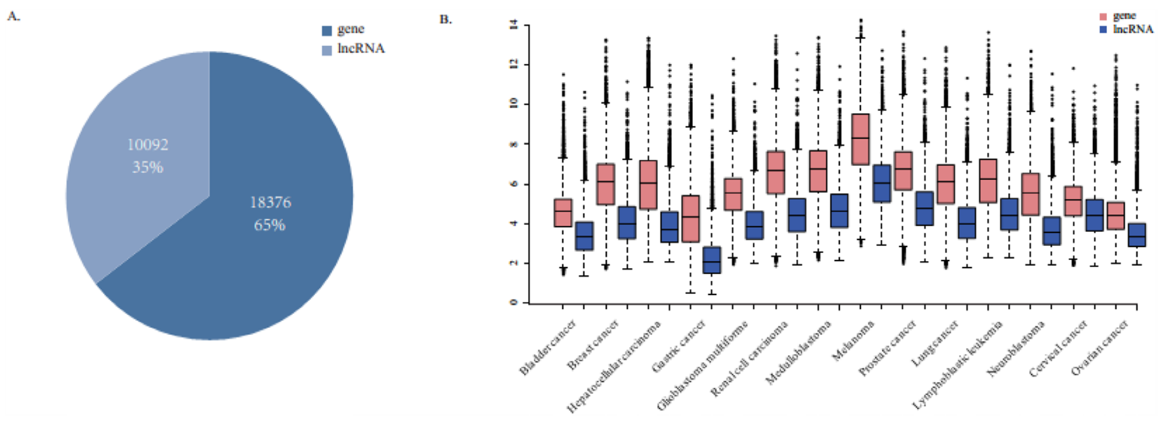

Supplementary Figure 1: The numbers of genes and lncRNAs in the array-based expression datasets. A. Pie chart representing the numbers of lncRNAs and genes. **B.** The box plots represent the expression levels of lncRNAs and genes in fourteen cancer types.

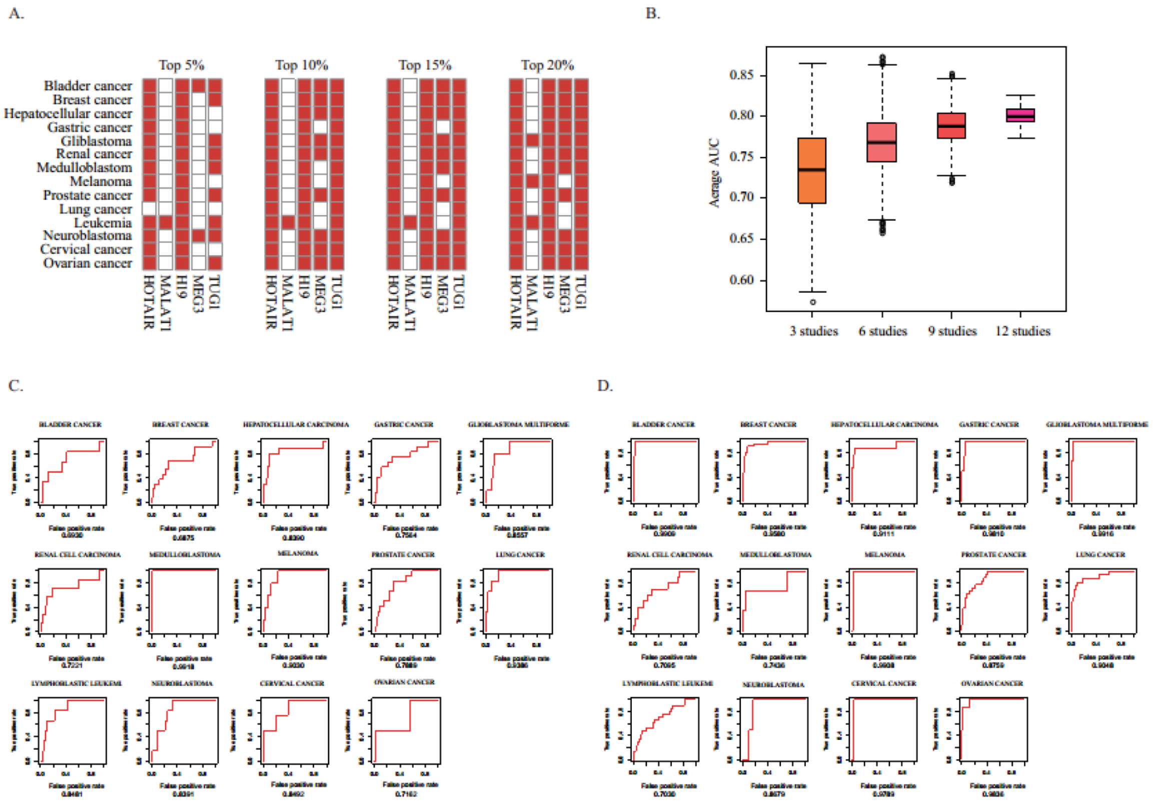

Supplementary Figure 2: Ranks of representative disease lncRNAs, results generated by randomly selected cancer types and prediction results. **A.** The ranks of five representative lncRNAs in the top 5%-20% of candidate lncRNA lists. **B.** The prioritization results through random selection of 3, 6, 9, and 12 cancer types. **C** and **D.** represent the gene and lncRNA prediction results calculated only using other disease information.

Supplementary Table 1: Array-based datasets of 14 cancer types

Cancer types	OMIM ID	Gse ID	Platform ID	NO. of samples
Bladder cancer	109800	GSE39016	GPL5188	141
		GSE41614	GPL5175	5
Breast cancer	114480	GSE33692	GPL5175	45
		GSE29044	GPL5175	8
		GSE16534	GPL5188	84
Hepatocellular carcinoma	114550	GSE12941	GPL5175	20
Gastric cancer	137215	GSE33429	GPL5175	25
		GSE30727	GPL5188	60
		GSE27342	GPL5175	80
		GSE13195	GPL5175	100
Glioblastoma multiforme	137800	GSE43388	GPL5188	95
		GSE24557	GPL5175	32
		GSE9385	GPL5188	49
Renal cell carcinoma	144700	GSE47032	GPL5175	20
Medulloblastoma	155255	GSE21166	GPL5175	90
		GSE21140	GPL5175	103
Melanoma	155601	GSE44662	GPL5175	38
Prostate cancer	176807	GSE42954	GPL5175	100
		GSE41410	GPL5175	48
		GSE30521	GPL9793	18
		GSE29079	GPL5175	47
		GSE21032	GPL5188	150
		GSE12378	GPL5175	36
Lung cancer	211980	GSE40275	GPL15974	42
		GSE37138	GPL5188	117
		GSE37058	GPL10200	52
		GSE22874	GPL5175	31
		GSE16534	GPL5188	43
		GSE12815	GPL5175	37
		GSE12236	GPL5188	20
		E-MEXP-2644	A-AFFY-143	17
Lymphoblastic leukemia	247640	GSE58211	GPL18761	300
		GSE43754	GPL5188	9
		GSE42731	GPL5175	50
		GSE35203	GPL5175	43
		GSE34186	GPL5175	15
		GSE30257	GPL5175	93
		GSE27370	GPL5188	162
Neuroblastoma	256700	GSE32664	GPL5175	87
		GSE27608	GPL5188	47
		GSE21713	GPL5175	40
Cervical cancer	603956	GSE39067	GPL5188	161
Ovarian cancer	604370	GSE29156	GPL5188	68
Total NO				2828

Supplementary Table 2: The numbers of nodes and edges in the GLCPN

Network	Nodes	Edges
GLCPN	29071	159132861
Bladder cancer	26656	32541329
Breast cancer	24314	9121233
Cervical cancer	505	3818
Gastric cancer	10686	152455
Glioblastoma multiforme	19779	8359247
Hepatocellular carcinoma	19233	404765
Lymphoblastic leukemia	25018	20418810
Lung cancer	25123	46881259
Medulloblastom	1279	5507
Melanoma	13288	828112
Neuroblastoma	8687	426624
Ovarian cancer	1940	18634
Prostate cancer	16236	1808554
Renal cell carcinoma	23826	19539494

Supplementary Table 3: Disease phenotype similarity scores of 14 cancers

OMIMID	109800	114480	114550	137215	137800	144700	155255	155601	176807	211980	247640	256700	603956	604370
**109800**	1	0.247168	0.34782	0.130349	0.148738	0.432695	0.174178	0.223959	0.305113	0.169745	0.085626	0.315231	0.528759	0.228698
**114480**	0.247168	1	0.301557	0.197589	0.270636	0.306267	0.23749	0.24059	0.510791	0.374483	0.270522	0.409273	0.267184	0.269652
**114550**	0.34782	0.301557	1	0.277286	0.209309	0.336462	0.234767	0.124763	0.345502	0.248505	0.08022	0.278069	0.297803	0.225959
**137215**	0.130349	0.197589	0.277286	1	0.191123	0.229745	0.420701	0.071357	0.208901	0.195782	0.088797	0.158627	0.242905	0.28791
**137800**	0.148738	0.270636	0.209309	0.191123	1	0.284124	0.39567	0.346235	0.247238	0.184844	0.141601	0.313741	0.118113	0.077111
**144700**	0.432695	0.306267	0.336462	0.229745	0.284124	1	0.285487	0.211106	0.267725	0.366454	0.216629	0.431997	0.252739	0.26164
**155255**	0.174178	0.23749	0.234767	0.420701	0.39567	0.285487	1	0.178215	0.231139	0.232245	0.146467	0.295711	0.218604	0.269393
**155601**	0.223959	0.24059	0.124763	0.071357	0.346235	0.211106	0.178215	1	0.113735	0.323511	0.168213	0.195604	0.17387	0.109171
**176807**	0.305113	0.510791	0.345502	0.208901	0.247238	0.267725	0.231139	0.113735	1	0.241548	0.195777	0.254873	0.36647	0.348211
**211980**	0.169745	0.374483	0.248505	0.195782	0.184844	0.366454	0.232245	0.323511	0.241548	1	0.186241	0.255317	0.130329	0.129691
**247640**	0.085626	0.270522	0.08022	0.088797	0.141601	0.216629	0.146467	0.168213	0.195777	0.186241	1	0.140822	0.091299	0.175811
**256700**	0.315231	0.409273	0.278069	0.158627	0.313741	0.431997	0.295711	0.195604	0.254873	0.255317	0.140822	1	0.225636	0.16275
**603956**	0.528759	0.267184	0.297803	0.242905	0.118113	0.252739	0.218604	0.17387	0.36647	0.130329	0.091299	0.225636	1	0.377004
**604370**	0.228698	0.269652	0.225959	0.28791	0.077111	0.26164	0.269393	0.109171	0.348211	0.129691	0.175811	0.16275	0.377004	1

Supplementary Table 4: Rank positions of three known disease genes on the top 20 disease candidate genes in GBM

Rank	Gene ID	Gene Symbol	Disease genes
1	3417	IDH1	known
2	2064	ERBB2	known
3	1029	CDKN2A	-
4	5468	PPARG	-
5	4830	NME1	-
6	23095	KIF1B	-
7	8643	PTCH2	-
8	51684	SUFU	-
9	2261	FGFR3	-
10	3845	KRAS	-
11	999	CDH1	-
12	5290	PIK3CA	-
13	4233	MET	-
14	3553	IL1B	-
15	3557	IL1RN	-
16	841	CASP8	-
17	5925	RB1	-
18	3265	HRAS	-
19	10460	TACC3	-
20	7157	TP53	known

Supplementary Table 5: GO enrichment result of seventeen unknown disease candidate genes

See Supplementary File 1

Supplementary Table 6: GO enrichment result of candidate lncRNAs listed on top 20

See Supplementary File 2
