# Supplementary material for: Systemically identifying and prioritizing risk lncRNAs through integration of pan-cancer phenotype associations
Source: Oncotarget. 2017 Jan 5;8(7):12041–51. doi: 10.18632/oncotarget.14510 (PMC5355324; doi:10.18632/oncotarget.14510)
Supplement: Supplementary file 2 [file oncotarget-08-12041-s002.doc]

**Supplementary table 5. GO enrichment result of seventeen unknown disease candidate genes.**

| Term | Count | P-Value | Benjamini | FDR |
| --- | --- | --- | --- | --- |
| GO:0010941 ~ regulation of cell death | 8 | 1.99E-05 | 0.003837059 | 0.03036 |
| GO:0043067 ~ regulation of programmed cell death | 4 | 4.03E-05 | 0.006212567 | 0.061509 |
| GO:0046822 ~ regulation of nucleocytoplasmic transport | 6 | 5.21E-05 | 0.006693087 | 0.079532 |
| GO:0051338 ~ regulation of transferase activity | 8 | 1.82E-05 | 0.00701107 | 0.027782 |
| GO:0042981 ~ regulation of apoptosis | 4 | 7.39E-05 | 0.008135249 | 0.112843 |
| GO:0032386 ~ regulation of intracellular transport | 7 | 1.13E-04 | 0.010911836 | 0.173169 |
| GO:0010033 ~ response to organic substance | 8 | 1.58E-05 | 0.012161012 | 0.024157 |
| GO:0042127 ~ regulation of cell proliferation | 4 | 1.81E-04 | 0.015478193 | 0.276836 |
| GO:0051348 ~ negative regulation of transferase activity | 4 | 2.71E-04 | 0.020775212 | 0.41369 |
| GO:0007568 ~ aging | 4 | 3.59E-04 | 0.024949928 | 0.547299 |
| GO:0051101 ~ regulation of DNA binding | 4 | 7.13E-04 | 0.028637107 | 1.084153 |
| GO:0051098 ~ regulation of binding | 5 | 7.04E-04 | 0.029817588 | 1.070154 |
| GO:0008285 ~ negative regulation of cell proliferation | 5 | 6.96E-04 | 0.031221551 | 1.059112 |
| GO:0060548 ~ negative regulation of cell death | 5 | 6.89E-04 | 0.032801121 | 1.04815 |
| GO:0043069 ~ negative regulation of programmed cell death | 3 | 6.78E-04 | 0.034382925 | 1.030956 |
| GO:0007569 ~ cell aging | 5 | 6.75E-04 | 0.036635132 | 1.026468 |
| GO:0043549 ~ regulation of kinase activity | 5 | 5.93E-04 | 0.037558494 | 0.902997 |
| GO:0045859 ~ regulation of protein kinase activity | 5 | 6.54E-04 | 0.038189379 | 0.994544 |
| GO:0043066 ~ negative regulation of apoptosis | 5 | 0.001174283 | 0.044453129 | 1.779731 |
| GO:0008284 ~ positive regulation of cell proliferation | 4 | 0.001375379 | 0.049462293 | 2.081525 |
| GO:0048545 ~ response to steroid hormone stimulus | 5 | 0.001818743 | 0.0570178 | 2.743842 |
| GO:0042325 ~ regulation of phosphorylation | 3 | 0.001744812 | 0.057074496 | 2.633692 |
| GO:0033157 ~ regulation of intracellular protein transport | 4 | 0.001683421 | 0.057553109 | 2.542136 |
| GO:0008361 ~ regulation of cell size | 4 | 0.00192801 | 0.05799888 | 2.906426 |
| GO:0042493 ~ response to drug | 5 | 0.002106063 | 0.060833023 | 3.170815 |
| GO:0051174 ~ regulation of phosphorus metabolic process | 5 | 0.002106063 | 0.060833023 | 3.170815 |
| GO:0019220 ~ regulation of phosphate metabolic process | 7 | 0.002238048 | 0.062210043 | 3.366365 |
| GO:0007242 ~ intracellular signaling cascade | 4 | 0.002481545 | 0.066376717 | 3.726163 |
| GO:0030097 ~ hemopoiesis | 4 | 0.002856562 | 0.073507899 | 4.277849 |
| GO:0060341 ~ regulation of cellular localization | 3 | 0.003022246 | 0.07512068 | 4.520644 |
| GO:0043388 ~ positive regulation of DNA binding | 4 | 0.00326502 | 0.078408812 | 4.875368 |
| GO:0048534 ~ hemopoietic or lymphoid organ development | 4 | 0.003942537 | 0.08142532 | 5.858804 |
| GO:0051094 ~ positive regulation of developmental process | 3 | 0.004943499 | 0.081707263 | 7.294356 |
| GO:0033673 ~ negative regulation of kinase activity | 3 | 0.004731532 | 0.081827229 | 6.992077 |
| GO:0051146 ~ striated muscle cell differentiation | 5 | 0.004856104 | 0.082067456 | 7.169837 |
| GO:0012501 ~ programmed cell death | 4 | 0.003902812 | 0.082843131 | 5.801405 |
| GO:0043086 ~ negative regulation of catalytic activity | 3 | 0.00526958 | 0.083331467 | 7.757576 |
| GO:0030099 ~ myeloid cell differentiation | 3 | 0.005159806 | 0.083363326 | 7.601876 |
| GO:0030308 ~ negative regulation of cell growth | 2 | 0.004723065 | 0.08354795 | 6.979983 |
| GO:0035022 ~ positive regulation of Rac protein signal transduction | 5 | 0.004181941 | 0.083932408 | 6.20403 |
| GO:0044093 ~ positive regulation of molecular function | 3 | 0.003737449 | 0.084078288 | 5.56212 |
| GO:0051099 ~ positive regulation of binding | 3 | 0.003737449 | 0.084078288 | 5.56212 |
| GO:0051384 ~ response to glucocorticoid stimulus | 4 | 0.004656305 | 0.084337324 | 6.884577 |
| GO:0009967 ~ positive regulation of signal transduction | 4 | 0.003863335 | 0.084347181 | 5.744333 |
| GO:0002520 ~ immune system development | 4 | 0.003669654 | 0.08508399 | 5.463855 |
| GO:0032535 ~ regulation of cellular component size | 3 | 0.004627181 | 0.085834592 | 6.842928 |
| GO:0006469 ~ negative regulation of protein kinase activity | 3 | 0.004421758 | 0.086310009 | 6.548663 |
| GO:0031960 ~ response to corticosteroid stimulus | 5 | 0.004605435 | 0.087540233 | 6.811818 |
| GO:0006915 ~ apoptosis | 3 | 0.006426197 | 0.091499765 | 9.383245 |
| GO:0051090 ~ regulation of transcription factor activity | 3 | 0.005950771 | 0.091756553 | 8.718284 |
| GO:0045792 ~ negative regulation of cell size | 4 | 0.006577879 | 0.091880403 | 9.594443 |
| GO:0044092 ~ negative regulation of molecular function | 4 | 0.006415598 | 0.09306075 | 9.36847 |
| GO:0051726 ~ regulation of cell cycle | 4 | 0.006308774 | 0.093323148 | 9.219432 |
| GO:0010647 ~ positive regulation of cell communication | 3 | 0.006305754 | 0.09509068 | 9.215215 |
| GO:0043406 ~ positive regulation of MAP kinase activity | 4 | 0.00696613 | 0.095341055 | 10.13294 |
| GO:0040008 ~ regulation of growth | 3 | 0.007557337 | 0.097882426 | 10.94717 |
| GO:0019216 ~ regulation of lipid metabolic process | 3 | 0.007427492 | 0.09791064 | 10.76894 |
| GO:0007611 ~ learning or memory | 3 | 0.007298682 | 0.097953335 | 10.59179 |
| GO:0045926 ~ negative regulation of growth | 3 | 0.007820125 | 0.099466943 | 11.30688 |
| GO:0051223 ~ regulation of protein transport | 3 | 0.007820125 | 0.099466943 | 11.30688 |
| GO:0010564 ~ regulation of cell cycle process | 5 | 0.008835103 | 0.101765708 | 12.68347 |
| GO:0016265 ~ death | 3 | 0.008772157 | 0.102594715 | 12.59869 |
| GO:0042692 ~ muscle cell differentiation | 3 | 0.008772157 | 0.102594715 | 12.59869 |
| GO:0070201 ~ regulation of establishment of protein localization | 4 | 0.008720177 | 0.103573516 | 12.52861 |
| GO:0007243 ~ protein kinase cascade | 5 | 0.00862427 | 0.104079738 | 12.39918 |
| GO:0008219 ~ cell death | 4 | 0.008591572 | 0.105338758 | 12.35501 |
| GO:0009628 ~ response to abiotic stimulus | 4 | 0.008527698 | 0.106270006 | 12.26867 |

KEGG pathway enrichment result of candidate genes listed on top 20.

| Term | Count | P-Value | Benjamini | FDR |
| --- | --- | --- | --- | --- |
| hsa05200:Pathways in cancer | 12 | 4.76E-12 | 3.24E-10 | 4.89E-09 |
| hsa05218:Melanoma | 7 | 9.50E-09 | 3.23E-07 | 9.78E-06 |
| hsa05219:Bladder cancer | 6 | 3.69E-08 | 8.36E-07 | 3.79E-05 |
| hsa05223 :Non-small cell lung cancer | 5 | 7.57E-06 | 1.29E-04 | 0.007786 |
| hsa05214:Glioma | 5 | 1.41E-05 | 1.91E-04 | 0.014478 |
| hsa05220:Chronic myeloid leukemia | 5 | 2.82E-05 | 3.20E-04 | 0.029039 |
| hsa05216:Thyroid cancer | 4 | 4.59E-05 | 4.46E-04 | 0.047229 |
| hsa05213:Endometrial cancer | 4 | 2.68E-04 | 0.002279304 | 0.275803 |
| hsa05211:Renal cell carcinoma | 4 | 6.47E-04 | 0.004880842 | 0.663994 |
| hsa05212:Pancreatic cancer | 4 | 7.03E-04 | 0.004772195 | 0.721101 |
| hsa05215:Prostate cancer | 4 | 0.001306 | 0.008044401 | 1.335162 |
| hsa05221:Acute myeloid leukemia | 3 | 0.009201 | 0.051030684 | 9.07114 |
