# Supplementary material for: Systemically identifying and prioritizing risk lncRNAs through integration of pan-cancer phenotype associations
Source: Oncotarget. 2017 Jan 5;8(7):12041–51. doi: 10.18632/oncotarget.14510 (PMC5355324; doi:10.18632/oncotarget.14510)
Supplement: Supplementary file 3 [file oncotarget-08-12041-s003.doc]

**Supplementary table 6.** **GO enrichment result of candidate lncRNAs listed on top 20.**

| GO term name | P-value | Corrected P-value |
| --- | --- | --- |
| transmission of nerve impulse | 1.76E-69 | 9.53E-65 |
| multicellular organismal signaling | 1.55E-68 | 4.20E-64 |
| synaptic transmission | 1.36E-66 | 2.45E-62 |
| cell-cell signaling | 2.44E-53 | 1.89E-49 |
| neurological system process | 8.44E-53 | 5.70E-49 |
| system process | 2.10E-51 | 1.26E-47 |
| single-multicellular organism process | 2.39E-34 | 4.78E-31 |
| multicellular organismal process | 4.92E-34 | 9.49E-31 |
| ion transport | 6.96E-31 | 1.14E-27 |
| nervous system development | 1.68E-27 | 2.22E-24 |
| neurotransmitter transport | 6.12E-25 | 7.51E-22 |
| single organism signaling | 3.78E-21 | 3.93E-18 |
| signaling | 3.78E-21 | 3.93E-18 |
| neurotransmitter secretion | 6.88E-21 | 6.89E-18 |
| cell communication | 1.55E-20 | 1.52E-17 |
| neuron differentiation | 3.72E-20 | 3.59E-17 |
| neurogenesis | 7.12E-20 | 6.63E-17 |
| generation of neurons | 1.49E-19 | 1.37E-16 |
| regulation of neurotransmitter levels | 1.52E-19 | 1.37E-16 |
| regulation of membrane potential | 1.10E-18 | 9.57E-16 |
| cation transport | 1.57E-18 | 1.35E-15 |
| single-organism transport | 5.39E-18 | 4.55E-15 |
| behavior | 8.25E-18 | 6.76E-15 |
| single-organism behavior | 1.02E-17 | 8.22E-15 |
| ion homeostasis | 1.12E-17 | 8.88E-15 |
| metal ion transport | 1.18E-17 | 9.22E-15 |
| signal release | 1.68E-17 | 1.28E-14 |
| generation of a signal involved in cell-cell signaling | 1.68E-17 | 1.28E-14 |
| monovalent inorganic cation transport | 4.14E-17 | 3.06E-14 |
| transport | 4.39E-17 | 3.21E-14 |
| cellular ion homeostasis | 6.20E-17 | 4.47E-14 |
| cellular chemical homeostasis | 6.54E-17 | 4.65E-14 |
| regulation of synaptic transmission | 8.33E-17 | 5.85E-14 |
| establishment of localization | 9.66E-17 | 6.69E-14 |
| cellular homeostasis | 4.45E-16 | 3.04E-13 |
| system development | 6.62E-16 | 4.41E-13 |
| regulation of neurological system process | 6.81E-16 | 4.49E-13 |
| chemical homeostasis | 1.12E-15 | 7.31E-13 |
| regulation of transmission of nerve impulse | 2.67E-15 | 1.70E-12 |
| neuron development | 3.91E-15 | 2.46E-12 |
| localization | 6.98E-15 | 4.33E-12 |
| regulation of biological quality | 7.53E-15 | 4.62E-12 |
| single-organism process | 4.26E-14 | 2.50E-11 |
| neuron projection development | 1.03E-12 | 5.46E-10 |
| anatomical structure development | 1.18E-12 | 6.17E-10 |
| multicellular organismal development | 1.53E-12 | 7.88E-10 |
| sodium ion transport | 1.86E-12 | 9.47E-10 |
| transmembrane transport | 3.01E-12 | 1.52E-09 |
| regulation of system process | 3.44E-12 | 1.70E-09 |
| membrane depolarization | 4.29E-12 | 2.11E-09 |
| learning or memory | 4.81E-12 | 2.34E-09 |
| cell development | 6.59E-12 | 3.18E-09 |
| secretion by cell | 1.90E-11 | 9.01E-09 |
| regulation of synaptic plasticity | 2.19E-11 | 1.03E-08 |
| G-protein coupled receptor signaling pathway | 4.19E-11 | 1.92E-08 |
| single-organism cellular process | 4.37E-11 | 1.98E-08 |
| developmental process | 4.80E-11 | 2.16E-08 |
| secretion | 5.61E-11 | 2.49E-08 |
| cognition | 6.81E-11 | 2.94E-08 |
| neuron projection morphogenesis | 7.70E-11 | 3.27E-08 |
| regulation of neuron differentiation | 7.86E-11 | 3.32E-08 |
| anion transport | 1.14E-10 | 4.64E-08 |
| regulation of neuron projection development | 1.22E-10 | 4.93E-08 |
| regulation of ion transport | 1.77E-10 | 7.04E-08 |
| central nervous system development | 1.89E-10 | 7.44E-08 |
| regulation of nervous system development | 3.42E-10 | 1.32E-07 |
| homeostatic process | 3.64E-10 | 1.40E-07 |
| cell morphogenesis involved in neuron differentiation | 4.11E-10 | 1.56E-07 |
| synapse organization | 4.41E-10 | 1.66E-07 |
| learning | 5.13E-10 | 1.93E-07 |
| synaptic vesicle transport | 5.71E-10 | 2.13E-07 |
| cell differentiation | 9.12E-10 | 3.31E-07 |
| regulation of glutamate receptor signaling pathway | 1.26E-09 | 4.53E-07 |
| potassium ion transport | 1.45E-09 | 5.14E-07 |
| regulation of alpha-amino-3-hydroxy-5-methyl-4-isoxazole propionate selective glutamate receptor activity | 3.30E-09 | 1.16E-06 |
| cell morphogenesis involved in differentiation | 4.44E-09 | 1.55E-06 |
| regulation of cell projection organization | 5.63E-09 | 1.94E-06 |
| regulation of neurogenesis | 6.13E-09 | 2.09E-06 |
| cellular developmental process | 1.09E-08 | 3.66E-06 |
| regulation of ion transmembrane transporter activity | 1.11E-08 | 3.71E-06 |
| excretion | 1.30E-08 | 4.30E-06 |
| cell projection organization | 1.48E-08 | 4.86E-06 |
| regulation of ion transmembrane transport | 1.53E-08 | 4.98E-06 |
| regulation of transporter activity | 2.18E-08 | 6.88E-06 |
| neuron-neuron synaptic transmission | 2.36E-08 | 7.41E-06 |
| axonogenesis | 2.48E-08 | 7.69E-06 |
| regulation of transmembrane transporter activity | 3.69E-08 | 1.13E-05 |
| cell projection morphogenesis | 4.65E-08 | 1.41E-05 |
| single-organism developmental process | 6.09E-08 | 1.84E-05 |
| regulation of transmembrane transport | 7.36E-08 | 2.19E-05 |
| regulation of postsynaptic membrane potential | 7.39E-08 | 2.19E-05 |
| synaptic vesicle exocytosis | 8.03E-08 | 2.37E-05 |
| regulation of excitatory postsynaptic membrane potential | 8.30E-08 | 2.44E-05 |
| regulation of transport | 1.09E-07 | 3.12E-05 |
| cell part morphogenesis | 1.09E-07 | 3.12E-05 |
| organic anion transport | 1.23E-07 | 3.46E-05 |
| glutamate secretion | 1.37E-07 | 3.78E-05 |
| regulation of cell morphogenesis involved in differentiation | 1.36E-07 | 3.78E-05 |
| regulation of dendrite development | 1.64E-07 | 4.51E-05 |
| regulation of synapse organization | 1.64E-07 | 4.51E-05 |
| locomotory behavior | 1.92E-07 | 5.22E-05 |
| gamma-aminobutyric acid signaling pathway | 2.69E-07 | 7.22E-05 |
| regulation of cell development | 2.94E-07 | 7.73E-05 |
| muscle organ development | 2.98E-07 | 7.82E-05 |
| cell morphogenesis | 4.53E-07 | 1.16E-04 |
| cell adhesion | 4.99E-07 | 1.26E-04 |
| biological adhesion | 5.60E-07 | 1.41E-04 |
| regulation of synapse structure and activity | 7.00E-07 | 1.74E-04 |
| exocytosis | 7.57E-07 | 1.87E-04 |
| regulation of exocytosis | 8.14E-07 | 2.00E-04 |
| synaptic vesicle endocytosis | 8.93E-07 | 2.18E-04 |
| dicarboxylic acid transport | 9.40E-07 | 2.28E-04 |
| cellular component morphogenesis | 9.66E-07 | 2.33E-04 |
| central nervous system neuron differentiation | 1.05E-06 | 2.51E-04 |
| amino acid transport | 1.15E-06 | 2.73E-04 |
| multicellular organismal response to stress | 1.26E-06 | 2.96E-04 |
| social behavior | 2.43E-06 | 5.61E-04 |
| ion transmembrane transport | 2.48E-06 | 5.71E-04 |
| neuromuscular process | 2.86E-06 | 6.50E-04 |
| regulation of neuronal synaptic plasticity | 2.88E-06 | 6.50E-04 |
| regulation of dendrite morphogenesis | 2.88E-06 | 6.50E-04 |
| regulation of multicellular organismal process | 3.72E-06 | 8.31E-04 |
| positive regulation of synaptic transmission | 5.09E-06 | 1.12E-03 |
| regulation of action potential | 5.36E-06 | 1.17E-03 |
| memory | 6.26E-06 | 1.36E-03 |
| positive regulation of neurological system process | 7.35E-06 | 1.59E-03 |
| neuron cell-cell adhesion | 7.43E-06 | 1.60E-03 |
| regulation of secretion | 7.77E-06 | 1.66E-03 |
| reproductive behavior | 7.77E-06 | 1.66E-03 |
| cell-cell adhesion | 8.62E-06 | 1.83E-03 |
| regulation of axonogenesis | 9.66E-06 | 2.03E-03 |
| positive regulation of transmission of nerve impulse | 1.07E-05 | 2.22E-03 |
| inorganic anion transport | 1.15E-05 | 2.38E-03 |
| vocalization behavior | 1.32E-05 | 2.72E-03 |
| multi-organism behavior | 1.35E-05 | 2.76E-03 |
| glutamate receptor signaling pathway | 1.41E-05 | 2.87E-03 |
| regulation of localization | 1.53E-05 | 3.08E-03 |
| single-organism reproductive behavior | 1.54E-05 | 3.09E-03 |
| regulation of synapse assembly | 1.68E-05 | 3.35E-03 |
| negative regulation of synaptic transmission | 2.04E-05 | 4.05E-03 |
| regulation of receptor activity | 2.17E-05 | 4.28E-03 |
| regulation of calcium ion-dependent exocytosis | 2.27E-05 | 4.45E-03 |
| fear response | 2.27E-05 | 4.45E-03 |
| regulation of cell morphogenesis | 2.72E-05 | 5.29E-03 |
| homophilic cell adhesion | 2.79E-05 | 5.39E-03 |
| behavioral defense response | 3.50E-05 | 6.54E-03 |
| behavioral fear response | 3.50E-05 | 6.54E-03 |
| neuron recognition | 4.07E-05 | 7.50E-03 |
| gliogenesis | 4.25E-05 | 7.81E-03 |
| neuronal action potential propagation | 4.35E-05 | 7.91E-03 |
| synaptic vesicle maturation | 4.35E-05 | 7.91E-03 |
| regulation of atrial cardiac muscle cell membrane depolarization | 4.35E-05 | 7.91E-03 |
| glial cell differentiation | 4.50E-05 | 8.16E-03 |
| brain development | 4.77E-05 | 8.61E-03 |
| carboxylic acid transport | 5.45E-05 | 9.75E-03 |
| organic acid transport | 5.45E-05 | 9.75E-03 |
| synaptic transmission, glutamatergic | 5.45E-05 | 9.75E-03 |

KEGG pathway enrichment result of candidate lncRNAs listed on top 20.

| Pathway name | P-value | Corrected P-value |
| --- | --- | --- |
| Neuronal System | 9.83E-35 | 1.69E-31 |
| Transmission across Chemical Synapses | 2.01E-25 | 1.72E-22 |
| Neuroactive ligand-receptor interaction | 1.47E-18 | 8.43E-16 |
| Nicotine addiction | 4.66E-15 | 2.00E-12 |
| Synaptic Vesicle Pathway | 4.58E-14 | 1.57E-11 |
| Transmembrane transport of small molecules | 4.16E-13 | 1.19E-10 |
| Neurotransmitter Receptor Binding And Downstream Transmission In The Postsynaptic Cell | 6.24E-13 | 1.53E-10 |
| Neurotransmitter Release Cycle | 1.80E-12 | 3.44E-10 |
| Retrograde endocannabinoid signaling | 1.71E-12 | 3.44E-10 |
| GABA synthesis, release, reuptake and degradation | 4.23E-12 | 7.25E-10 |
| Synaptic vesicle cycle | 3.00E-11 | 4.68E-09 |
| Potassium Channels | 4.89E-11 | 6.99E-09 |
| GABAergic synapse | 5.37E-11 | 7.08E-09 |
| SLC-mediated transmembrane transport | 8.25E-10 | 1.01E-07 |
| Acetylcholine Neurotransmitter Release Cycle | 1.31E-08 | 1.40E-06 |
| Acetylcholine Neurotransmitter Release Cycle | 1.31E-08 | 1.40E-06 |
| Neurotransmitter Release Cycle | 2.87E-08 | 2.90E-06 |
| Transport of inorganic cations/anions and amino acids/oligopeptides | 3.19E-08 | 3.04E-06 |
| Ligand-gated ion channel transport | 6.26E-08 | 5.66E-06 |
| Na+/Cl- dependent neurotransmitter transporters | 1.37E-07 | 1.13E-05 |
| Transport of glucose and other sugars, bile salts and organic acids, metal ions and amine compounds | 1.38E-07 | 1.13E-05 |
| Glutamate Neurotransmitter Release Cycle | 3.69E-07 | 2.54E-05 |
| Norepinephrine Neurotransmitter Release Cycle | 3.69E-07 | 2.54E-05 |
| GABA A receptor activation | 3.69E-07 | 2.54E-05 |
| Norepinephrine Neurotransmitter Release Cycle | 3.69E-07 | 2.54E-05 |
| GPCR Dopamine D1like receptor | 5.32E-07 | 3.51E-05 |
| Serotonin Neurotransmitter Release Cycle | 8.97E-07 | 5.50E-05 |
| Dopamine Neurotransmitter Release Cycle | 8.97E-07 | 5.50E-05 |
| Morphine addiction | 1.01E-06 | 5.99E-05 |
| Interaction between L1 and Ankyrins | 1.09E-06 | 6.25E-05 |
| Serotonin Neurotransmitter Release Cycle | 1.29E-06 | 6.70E-05 |
| Neurotransmitter Release Cycle | 1.29E-06 | 6.70E-05 |
| Dopamine Neurotransmitter Release Cycle | 1.29E-06 | 6.70E-05 |
| Voltage gated Potassium channels | 1.65E-06 | 8.33E-05 |
| GABA receptor activation | 2.51E-06 | 1.19E-04 |
| Ion channel transport | 2.51E-06 | 1.19E-04 |
| Benzodiazepine Pathway, Pharmacodynamics | 3.91E-06 | 1.81E-04 |
| Glycoprotein hormones | 4.32E-06 | 1.95E-04 |
| Amine compound SLC transporters | 6.93E-06 | 3.05E-04 |
| Dopaminergic synapse | 8.20E-06 | 3.52E-04 |
| Calcium Regulation in the Cardiac Cell | 1.02E-05 | 4.25E-04 |
| Glial Cell Differentiation | 1.21E-05 | 4.95E-04 |
| Glutamate Neurotransmitter Release Cycle | 1.29E-05 | 5.15E-04 |
| Glutamatergic synapse | 2.20E-05 | 8.59E-04 |
| Effects of Botulinum toxin | 2.84E-05 | 1.08E-03 |
| Calcium signaling pathway | 2.96E-05 | 1.10E-03 |
| Transport of glucose and other sugars, bile salts and organic acids, metal ions and amine compounds | 3.13E-05 | 1.14E-03 |
| Trafficking of AMPA receptors | 4.07E-05 | 1.42E-03 |
| Glutamate Binding, Activation of AMPA Receptors and Synaptic Plasticity | 4.07E-05 | 1.42E-03 |
| Peptide hormone biosynthesis | 4.94E-05 | 1.70E-03 |
| Hormone ligand-binding receptors | 5.85E-05 | 1.93E-03 |
| Na+/Cl- dependent neurotransmitter transporters | 5.85E-05 | 1.93E-03 |
| Prolactin receptor signaling | 8.21E-05 | 2.66E-03 |
| GPCR ligand binding | 8.43E-05 | 2.68E-03 |
| Activation of NMDA receptor upon glutamate binding and postsynaptic events | 9.55E-05 | 2.98E-03 |
| Unblocking of NMDA receptor, glutamate binding and activation | 1.31E-04 | 4.00E-03 |
| Organic anion transport | 1.48E-04 | 4.46E-03 |
| Organic cation/anion/zwitterion transport | 1.90E-04 | 5.54E-03 |
| Glycoprotein hormones | 1.90E-04 | 5.54E-03 |
| Amine ligand-binding receptors | 1.98E-04 | 5.66E-03 |
| Sympathetic Nerve Pathway (Pre- and Post- Ganglionic Junction) | 2.30E-04 | 6.46E-03 |
| Ras activation uopn Ca2+ infux through NMDA receptor | 2.96E-04 | 8.19E-03 |
| Axon guidance | 3.37E-04 | 9.18E-03 |
| Serotonergic synapse | 3.47E-04 | 9.30E-03 |
| GPCR signaling-cholera toxin | 3.63E-04 | 9.59E-03 |
